# The Crepitant or Vesicular Rale an Inter-pleural Sound and Not Caused in the Pulmonary Tissue*Read before the Medical Association of Georgia, Macon, Ga., April, 1897.

**Published:** 1897-08

**Authors:** Louis H. Jones

**Affiliations:** Atlanta, Ga.


### THE CREPITANT OR VESICULAR RALE AN INTERPLEURAL SOUND AND NOT CAUSED IN THE PULMONARY TISSUE.*

By LOUIS H. JONES, M. D., ATLANTA, GA.

Even among the most intelligent and truthful of our patients, we can often attach but little importance to subjective symptoms; we are forced to rely for a correct knowledge and accurate diagnosis of a case, upon such signs as we can elicit by a physical examination. If we are to draw correct conclusions, our methods must be accurate, our deductions must be logical; they must stand the test of careful scientific analysis; they must conform to known physical laws.

Believing that in our text-books on physical diagnosis, and in the lectures of a majority of teachers of this branch, that certain sounds heard in connection with diseases of the lungs are misinterpreted, I have thought it well to lay before the

*Read before the Medical Association of Georgia, Macon, Ga , April, 1897.

Association some of the views held by a most distinguished physician, now dead, and to add to his testimony the results of my own limited experience. Particularly do I desire to do this in regard to the origin, seat, and cause of what is known as the crepitant or vesicular rale. First let us see what the recognized authorities say: DaCosta, in speaking of the vesicular or crepitant rale, says: "Its name indicates its seat. It is caused by agitation of fluid in the air-cells, or in the finest extremities of the bronchial tubes; or, to adopt a view now held by many, by the forcing open during inspiration of the air-cells agglutinated by exuded lymph. The first stage of pneumonia is the state in which this rale is mostly engendered." Again, in speaking of pleural friction, he says: "It may be so much like crepitation, that even long practice in auscultation will not enable us to determine at once whether the fine sounds we hear are the friction of a roughened pleura, or the vesicular rales of an inflamed lung."

Loomis says of crepitant rales: "There are several views as to the production of these sounds; that they are due to the bubbling of air through a liquid in the pulmonary vesicles and terminal bronchioles; that at the end of expiration, a viscid secretion glues the walls of the vesicles together, and their separation on inspiration gives rise to the crackling noise; and that they are produced in the pleuræ independently of the pulmonary parenchyma."

Osler, in speaking of pneumonia, says: "Very early there is heard at the end of inspiration the fine crepitant rale, a series of minute cracklings heard close to the ear, and perhaps not audible until a full breath is drawn; whether this is a fine pleural crepitus or is produced in the air-ceils and finer bronchi, is an open question."

In speaking of pleurisy, Osler says: "There is another pleural friction sound which closely resembles, and is scarcely to be distinguished from the fine crackling crepitus of pneumonia. This may be heard at the commencement of the disease, and also, as pointed out by McDonell, Sr., of Montreal, when the fluid has receded and the pleural layers have come together again."

Page says: "Crepitant rales are made in the air-cells, and are the only vesicular rales that exist. They are very fine, uniform crackling rales, heard only at the (tip) end of inspiration and are unchanged by coughing. They are caused by the forcible separation of agglutinated cell-walls, as in the first stage of lobar pneumonia, or agitation of thin fluid in the air-cells, as in pulmonary edema. They are heard also in the third or resolving stage of pneumonia, where it is known as the rale redux, or rale that has returned or come back."

These expressions of opinion from the men who have stood at the top as diagnosticians for the past quarter of a century may, we think, be fairly taken as an expression of the most generally accepted belief concerning the cause and location of the crepitant rale; and while the opinions quoted show that most of the authorities admit the possibility, and even assert the existence, during pleurisy, of an interpleural sound not to be distinguished from the crepitant rale, yet they believe and teach that what is called the crepitant rale, is a sound produced in the vesicles, and that it is indicative of a diseased condition of the pulmonary parenchyma.

Dr. James R. Leaming, in papers read before the New York Academy of Medicine more than twenty years ago, made the assertion that both the crepitant and the sub-crepitant rale were sounds which had their origin in the pleural, and not the pulmonary tissues. This position he maintained most ably, and claimed that he had repeatedly demonstrated its correctness by post-mortem examinations. Early in my practice I read Leaming's work on the heart and lungs, and was so impressed with his clear and logical reasoning, that I began questioning the correctness of what I had been taught as a medical student, and, as I had opportunity, I attempted to verify clinically his position.

Let us first look a moment at the structure of the lungs and the mechanism of respiration. The lungs are made up of tubes and of air-cells or vesicles; the tubular or convective portion consisting of tubes which gradually grow smaller and smaller until they become microscopic, and terminate in the air-cells. The cellular portion of the lung is capable of great distention, and is always completely filled with air; the volume of this air may be argumented by the expansion of the cells; it may be diminished by their contraction, but the cells must always be full, they can not be alternately full and empty. If partly filled with liquid, then air must occupy the remaining space. Were this not true, collapse of the lungs would inevitably occur, for the lung is always on the stretch, pulling, it is computed with an elastic force of one-fourth to one pound to the square inch, and thus tending to separate the pleuræ and close the air-cells; but, on the other hand, the air pressure on the interior of the lungs, that is, in the air-cells, is fifteen pounds to the square inch; now that such a discrepancy in pressure can be overcome, and the cells closed by their elastic force, is inconceivable; it is a physical impossibility. The lung is constantly being forcibly pressed by atmospheric pressure against the inside of the chest wall, thus completely filling the cavity of the thorax, whether in inspiration or expiration. If the cell-walls come together, the parietal and visceral pleuræ must separate. What then becomes of the theory of the alternate separation and apposition of the cell-walls covered with an exuded lymph?

Now let us look at the other theory, viz.: that the crepitant rale is a sound caused by the bubbling of air through a liquid in the air-cells or terminal bronchioles. In order that air passing through a liquid should cause bubbling, it must move with some degree of rapidity, there must be a motion of the air in mass. Physiologists tell us that the air which is in contact with the respiratory epithelium, is not sucked in and out with each inspiration, but that the air-cells are constantly filled with the residual air, which constitutes about eight-tenths of the total air capacity of the lungs. The tidal air entering the larger bronchi with each inspiration, meets in the bronchioles the residual air and its current is checked, motion in mass ceases, and molecular motion takes its place, a rapid diffusion of gases occurs, the tidal air yielding up its oxygen and getting in exchange the carbon dioxide of the residual air. The conditions for such diffusion are well fulfilled in the lungs, and added to this we have the red corpuscles exerting their eager affinity for the oxygen, and thus hastening the change. We all know that under such conditions diffusion will be rapid, yet it will be only a molecular motion. Can we conceive it otherwise? Could it be possible that the tidal air could enter microscopic tubes and cells, overcoming the friction and displacing in mass a quantity of residual air eight to ten times its own volume? Is not such a supposition opposed to all known physical laws? Is it not at variance with all physiology? Must we not abandon the idea of air currents in the vesicles, causing bursting of bubbles? Was not the theory set up to meet supposed conditions rather than to accord with known laws?

Pathology teaches us that early in pneumonia there is an exudation into the air-cells. Experience has shown us that early in the same disease there is usually heard the crepitant rale; thus the two things became to be associated together, without careful investigation and thought.

Unquestionably there are cases of pneumonia in which the crepitant rale is absent during the stage of exudation, but is clearly and umistakably present after complete consolidation has occurred. If this be true, the seat of the rale can not be in the vesicles.

Time will not allow me to go into a detailed report of cases, but within the last few months I have seen two cases of pneumonia classical in every respect save in the absence of the crepitant rale until *after* complete consolidation was present. The high temperature, cough, rusty sputa, complete consolidation, tubular breathing, were all present, yet no crepitant rale could be detected in the early stages, but after consolidation, the so-called vesicular rales were abundant. With the appearance of the rales, both patients began to complain of pain, the pain corresponding in location with the area over which the rales could be most distinctly heard. We well know that very few cases of pneumonia, or indeed of any serious lesion of the lungs, occur which are not accompanied by an involvement of the pleuræ. If, then, we reason, *post hoc ergo propter hoc*, have we not both in pneumonia and in pulmonary phthisis the same ground for locating the crepitant rale in the pleuræ that we have for placing it in the pulmonary parenchyma? But are not the physical conditions for the production of the sound under discussion present, where we have inflamed pleural surfaces covered with a fibrous exudation, moving the one over the other, constantly attempting to separate, yet ever being held together by atmospheric pressure?

The human ear can locate with great accuracy the distance of a sound. Osler says of the crepitant rale: "A series of minute cracklings heard close to the ear." This is always true of the sound under discussion; it lacks the element of distance. While not a loud sound, yet the ear placed in contact with the naked chest wall, will recognize that it hears a sound which is immediately beneath the surface, and not produced deep down in the lung tissue. The ear takes cognizance of distance, just as a muscle distinguishes between a light and a heavy load.

Careful and oft-repeated physical examinations, together with post-mortems when possible, must determine the question under discussion. We appeal to the profession to make such investigation, and not to accept without question the teachings of the day.

### DISCUSSION OF DR. JONES'S PAPER.

DR. J. W. DUNCAN, Atlanta: As no one else manifests a desire to speak, I will say a few words in reference to Dr. Jones's paper. In a physiological condition of the lungs we hear the normal vesicular murmur. While I have very great regard for the doctor's opinion on any medical subject, yet he did not mention that fact. He did not explain the vesicular murmur in normal lung tissue. He did not give us the idea whence that vesicular murmur originates, where it is formed, whether in the bronchioles or air-cells, or the larger bronchial tubes. He also mentions the fact that in the fine crepitation of pneumonia the sound was very near the ear. If this sound was produced even in the center of the lung tissue, or near it, or near the surface, there being some consolidation of lung tissue, it would naturally favor the sound being close to the ear, a solid body being a better conductor of sound than air itself. For a long time I have been taught and have believed that this murmur takes place in the bronchioles, and not strictly in the air-cells, as some have claimed. It is hard for me to accept any other view, and in the latter stages of pneumonia, as the disease is declining and resolution taking place, we have a different crepitant rale, a sub-crepitant rale, a louder sound; some call it a moist sound. I am satisfied that it is the larger bronchial tubes and the bronchioles. I think the nearness of the sound in a true fine crepitant rale in pneumonia, appearing to be so close to the ear, is not proof that it is intrapleural, but that it is in consequence of solidified lung tissue, which makes it a better conductor. You hear the whispering bronchophony in pneumonia, and you hear the noise in the throat of the patient while your ear is to the chest, being communicated from the throat to the ear. I think I have seen cases of pneumonia without involvement of the pleura. I have heard of the crepitant rale in cases where the pleura was not involved, where the patient had no pleuritic pain, nor any indications of pleurisy, unless the crepitant rale is an indication; consequently I have been inclined heretofore to differentiate my cases of pleuro-pneumonia and pneumonia.

DR. JONES (closing the discussion): Dr. Duncan has said that I did not explain the cause of normal vesicular murmur. I did not undertake to do it in the limited time at my disposal, and particularly to dwell upon the various theories as to the cause of the normal vesicular murmur, among which are muscular contraction and a number of others. I was simply endeavoring to explain my views as to the location of the crepitant rale.

